# Tyrosine-protein phosphatase non-receptor type 12 expression is a good prognostic factor in resectable non-small cell lung cancer

**DOI:** 10.18632/oncotarget.3588

**Published:** 2015-03-14

**Authors:** Xun Cao, Yan-Zhen Chen, Ruo-Zhen Luo, Lin Zhang, Song-Liang Zhang, Jun Zeng, Yu-Chuan Jiang, Yu-Jing Han, Zhe-Sheng Wen

**Affiliations:** ^1^ Sun Yat-Sen University Cancer Center, State Key Laboratory of Oncology in South China, Collaborative Innovation Center of Cancer Medicine, Guangzhou, Guangdong, China; ^2^ Department of Pathology, Sun Yat-Sen University Cancer Center, Guangzhou, Guangdong, China; ^3^ Department of Clinical Laboratory, Sun Yat-Sen University Cancer Center, Guangzhou, Guangdong, China; ^4^ Department of Preclinical Medicines, Sun Yat-Sen University Cancer Center, Guangzhou, Guangdong, China; ^5^ Department of Thoracic Oncology, Sun Yat-Sen University Cancer Center, Guangzhou, Guangdong, China

**Keywords:** non-small cell lung cancer, tyrosine-protein phosphatase non-receptor type 12, survival, prognosis

## Abstract

Tyrosine-protein phosphatase non-receptor type 12 (PTPN12) has been considered to be a tumor suppressor in human cancer, but its clinical and prognostic significance in non-small cell lung cancer (NSCLC) has not been well elucidated.

A retrospective analysis of 215 patients with surgically resected NSCLCs from Sun Yat-Sen University Cancer Center between April 2002 and March 2005 was performed using immunohistochemistry and Western Blot to analyze PTPN12 expression. The association between PTPN12 expression and patient survival was investigated.

Western Blots showed that the expression level of PTPN12 were higher in normal paracancerous lung tissues than in NSCLC tissues. High PTPN12 expression was less common in the presence than in the absence of visceral pleural invasion (p=0.038). Patients with PTPN12-high tumors had a longer disease-free survival (DFS) (*P*<0.001) and overall survival (OS) (p<0.001), especially for those with non-squamous cell carcinoma (non-SCC) (DFS, p<0.001; OS, p<0.001). Multivariate analysis confirmed that PTPN12 positivity was associated with increased survival duration (DFS, p<0.001; OS, p<0.001), independent of prognostic indicator.

High PTPN12 expressive levels are associated with favorable survival duration in patients with NSCLC, especially those with non-SCC. Our study suggests that PTPN12 expression is a valuable prognostic biomarker for NSCLC patients.

## INTRODUCTION

Lung cancer continue to be the most common causes of cancer death worldwide [1]. Patients with non-small cell lung cancer (NSCLC), which account for 75%-80% of lung cancer cases, carry a 5-years survival rate of 10%-15% for all stage. Curative-intent resection remains the mainstay of treatment and provides the best chance for survival [2]. Unfortunately, however, many patients with the same pathological Tumor-Node-Metastasis (TNM) stage have different survivals. Thus, the American Joint Committee on Cancer Staging system (AJCC) may be not reliable to predict clinical outcomes. Studies conducted several years ago have suggested that cancer has been envisioned as a signaling disease [3]. A better understanding of the biochemical signaling molecules and genetic factors of NSCLC could provide valuable prognostic parameter, improve the prognoses of patients and provide therapies that are more appropriate [4-7].

Protein tyrosine phosphatases (PTPs) are a group of enzymes that remove phosphate groups from phosphorylated tyrosine residues on proteins. In the past few years, several investigations implicated that PTPs are key regulatory components in signal transduction pathways, such as the MAP kinase pathway, and are important in the control of cell growth, cellular proliferation, cellular differentiation, mitotic cycles and oncogenic transformation [8-11]. Sun et al. have recently uncovered a new molecular biomarker, Tyrosine-Protein Phosphatase Non Receptor Type 12 (PTPN12), which is a member of the PTPs family [12]. PTPN12 can suppress cell proliferation and tumorigenicity by inhibiting multiple receptor tyrosine kinases (RTKs). RTKs regulate cell survival and proliferation and signal via autophosphorylation and recruitment of additional substrates through recognition of these autophosphorylation sites [9]. Activation of PTPN12 suppresses RTKs, leading to inhibition of cell growth, proliferation, transformation and tumorigenesis through downstream signaling. These functions implied that PTPN12 play a prominent role in tumor suppression.

However, the role of PTPN12 and the association between PTPN12 and survival in NSCLC have not been rigorously and systematically elucidated. Based on these considerations, the primary aim of the current study was to identify the expression of PTPN12 in NSCLC samples in a large patients cohort. In addition, the overarching goal of this research is to discuss the clinicopathological and predictive value of PTPN12 expression in NSCLC patients.

## RESULTS

### Patient characteristics

After exclusion of the non-informative samples (unrepresentative samples, samples with too few tumor cells and lost samples), PTPN12 expression was evaluated in a total of 215 (89.6%) patients. All further statistical analyses were performed on this population.

The median age of the patients was 60 years (range, 30 to 79 years), and 74.4% were men. Of 215 NSCLC patients, 76 (35.3%) had squamous cell carcinoma (SCC), and 139 (64.7%) had non-SCC. Table 1 summarizes the characteristics of the study population.

**Table 1:** PTPN12 expression and clinicopathological characteristics of the patients with NSCLCs

		PTPN12 expression (%)		
Characteristic	No. Patients (%)	Low	High	p Value^a^
Total	215	109 (50.7)	106 (49.3)	
Age (years) ^b^				0.090
≤ 60	114 (53.0)	64 (56.1)	50 (43.9)	
> 60	101 (47.0)	45 (44.6)	56 (55.4)	
Gender				0.556
Male	160 (74.4)	83 (51.9)	77 (48.1)	
Female	55 (25.6)	26 (47.3)	29 (52.7)	
Tumor laterality				0.792
Left	81 (37.7)	42 (51.9)	39 (48.1)	
Right	134 (62.3)	67 (50.0)	67 (50.0)	
Primary lobe				0.055
Upper lobe	102 (47.4)	43 (42.2)	59 (57.8)	
Middle lobe	21 (9.8)	13 (61.9)	8 (38.1)	
Lower lobe	92 (42.8)	53 (57.6)	39 (42.4)	
Histology				0.662
SCC	76 (35.3)	37 (48.7)	39 (51.3)	
Non-SCC^c^	139 (64.7)	72 (51.8)	67 (48.2)	
Visceral pleural invasion				0.038
Absent	63 (29.3)	25 (39.7)	38 (60.3)	
Present	152 (70.7)	84 (55.3)	68 (44.7)	
Tumor grade				0.264
Grade 1	28 (13.0)	18 (64.3)	10 (35.7)	
Grade 2	88 (40.9)	41 (46.6)	47 (53.4)	
Grade 3	99 (46.0)	50 (50.5)	49 (49.5)	
pT status				0.191
pT1	39 (18.1)	15 (38.5)	24 (61.5)	
pT2	150 (69.8)	81 (54.0)	69 (46.0)	
pT3	19 (8.8)	8 (42.1)	11 (57.9)	
pT4	7 (3.3)	5 (71.4)	2 (28.6)	
pN status				0.226
pN0	115 (53.5)	52 (45.2)	63 (54.8)	
pN1	44 (20.5)	25 (56.8)	19 (43.2)	
pN2	56 (26.0)	32 (57.1)	24 (42.9)	
pTNM stage				0.038
I	86 (40.0)	35 (40.7)	51 (59.3)	
II	67 (31.2)	41 (61.2)	26 (38.8)	
III	62 (28.8)	33 (53.2)	29 (46.8)	

Abbreviation: PTPN12, tyrosine-protein phosphatase non-receptor type 12; NSCLC, non-small cell lung cancer; SCC, squamous cell carcinoma.

a χ^2^ test;

b Median age;

c Non-SCC includes adenocarcinoma, adenosquamous carcinoma, anaplastic large-cell carcinoma, sarcoma, adenoid cystic carcinoma, mucoepidermoid carcinoma and carcinoid tumor.

### Western blot assessment of PTPN12 expression

**Figure 1:**
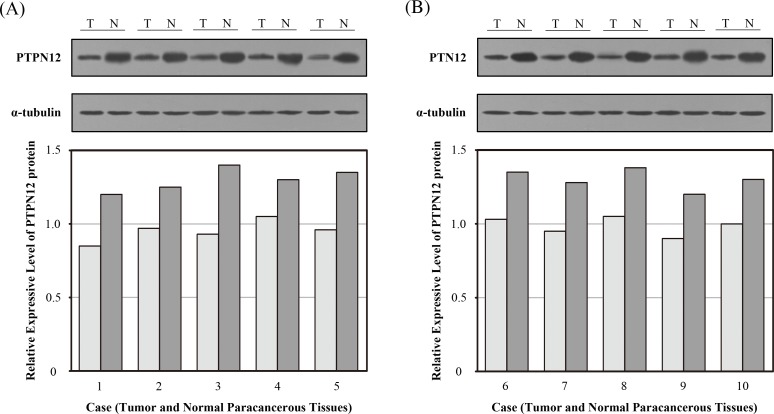
Expression of tyrosine-protein phosphatase non-receptor type 12 (PTPN12) in surgically resected lung tissues was determined by Western blot Expression of PTPN12 in normal paracancerous tissues and non-small cell lung cancer (NSCLC) tissues. The relative expression of PTPN12 in comparison to the expression level of α-tubulin. (A) Case 1-5; (B) Case 6-10. (T, tumor tissue; N, normal parecancerous tissue).

In order to ensure the reliability of this study, we first investigated the expression level of PTPN12 protein by Western blot in 10 pairs of surgically resected lung tissues. The relative PTPN12 expression was compared in NSCLC tissues and normal paracancerous tissues. Western blot analysis showed that the PTPN12 protein expression was higher in normal paracancerous tissue than in NSCLC tissues (Figure 1).

### Immunohistochemical assessment of PTPN12 expression

**Figure 2:**
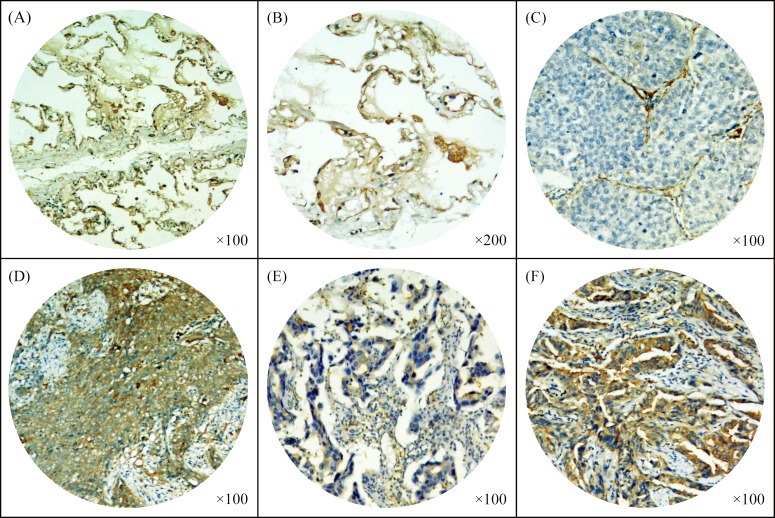
Immunohistochemical staining of tyrosine-protein phosphatase non-receptor type 12 (PTPN12) in paracancerous normal lung tissues and non-small cell lung cancer (NSCLC) tissues (A-B) Expression of PTPN12 in paracancerous normal lung tissues; (C-F) Expression of PTPN12 in squamous cell carcinoma (SCC) and adenocarcinoma (ADC): (C) A PTPN12-low expressive SCC tissue and (D) a PTPN12-high expressive SCC tissue; (E) A PTPN12-low expressive ADC tissue and (F) a PTPN12-high expressive ADC tissue.

Figure 2 showed that PTPN12 was localized to the cytoplasm. Using the criteria described above, of the 215 tumors, 106 (49.3%) were PTPN12-high expression, and 109 (50.7%) were PTPN12-low expression. Table 1 compared the clinicopathological parameters according to the expressive level of PTPN12. χ^2^ test showed that the PTPN12 expression was closely correlated with visceral pleural invasion (p=0.038, less common in the presence than in the absence of visceral pleural invasion), and pTNM stage (p=0.038, less common in pTNM stage II and III patients than pTNM stage I patients).

### PTPN12 expression and survival

The median follow-up period was 46 months with 89 alive and 126 cancer-related deaths at the last clinical follow-up. The 1-, 3-, 5-year survival rates for the entire cohort of patients were 69%, 49%, and 39%, respectively.

**Figure 3:**
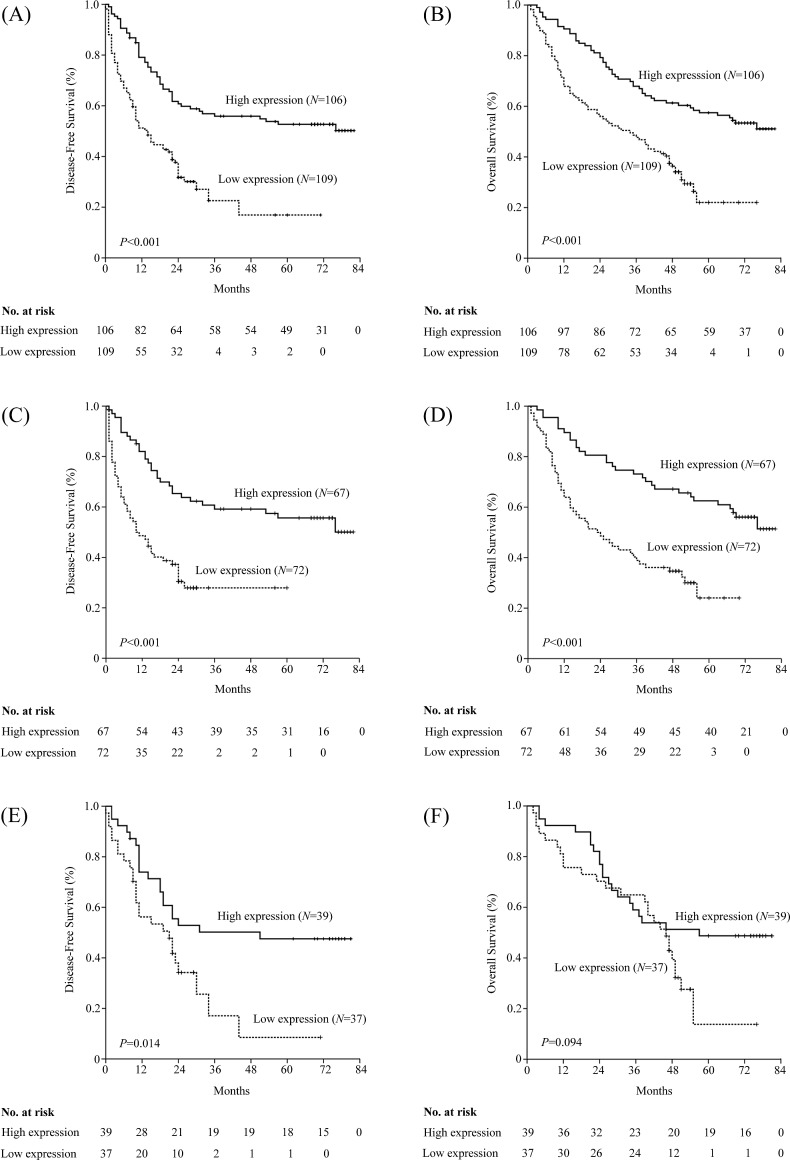
Kaplan–Meier estimates of the probability of survival in patients with non-small cell lung cancer (NSCLC) (A) Disease-free survival (DFS) and (B) overall survival (OS) curves for the whole cohort of patients with NSCLC; (C) DFS and (D) OS curves for the non-squamous cell carcinoma (non-SCC) patients; (E) DFS and (F) OS curves for the SCC patients.

In the Kaplan-Meier analysis, PTPN12 expression was significantly associated with DFS and OS. For the whole cohort, median DFS was longer in patients with high-PTPN12 expression than those with low-PTPN12 expression (p<0.001). Median OS among patients with PTPN12-high tumors was also longer than those with PTPN12-low tumors (p<0.001). Furthermore, we examined the relationship between PTPN12 expression and survival based upon histology subgroup. We observed a significant different in both DFS and OS durations in non-SCC patients with high or low-PTPN12 expression (DFS: 32 vs. 11 months, p<0.001; OS: 63 vs. 24 months, p<0.001), whereas OS durations were similar between high and low-PTPN12 expression subsets in patients with SCC (57 vs. 46 months, p=0.094). Details were showed in Table 2 and Figure 3.

**Table 2:** Prognostic significance of PTPN12 expression in patients with NSCLC

		Disease-Free Survival			Overall Survival		
PTPNI2 Expression	Number of Patients	Mean	Median	p Value^a^	Mean	Median	p Value^a^
Total				<0.001			<0.001
Low expression	109	23.5	14.0		36.1	34.0	
High expression	106	51.0	NR		57.1	NR	
Histology							
SCC				0.014			0.094
Low expression	37	22.7	21.0		39.5	46.0	
High expression	39	46.8	51.0		52.9	57.0	
Non-SCC				<0.001			<0.001
Low expression	72	22.9	10.0		32.5	23.0	
High expression	67	53.0	10		59.3	NR	

Abbreviation: PTPN12, tyrosine-protein phosphatase non-receptor type 12; NSCLC, non-small cell lung cancer; SCC, squamous cell carcinoma; ADC, adenocareinoma; NR, not reach: pT status, pathological tumor. pN status, pathological node; pTNM stage, pathological tumor-node-metastasis stage.

a Log-rank test.

To determine whether PTPN12 expression could serve as an independent prognostic parameter, we examined DFS and OS using the Cox proportional hazards model. The results revealed that pTNM stage (DFS: p<0.001; OS: p<0.001) and PTPN12 expression (DFS: p<0.001; OS: p<0.001) were independent, significant predictors for DFS and OS. Interestingly, when we carried out the multivariate analyses in each histology subgroup, the benefit of high-PTPN12 expression was especially significant in non-SCC patients (DFS: p<0.001; OS: p<0.001). The details are presented in Table 3.

**Table 3:** Multivariate Cox regression analysis for Disease-free Survival and Overall Survival in patients with NSCLC

		Disease-Free Survival		Overall Survival	
Cohort	Variables	HR (95%CI)	p Value^a^	HR (95%CI	p Value^a^
All population	pTNM Stage		<0.001		<0.001
	Stage I	Reference		Reference	
	Stage II	3.148 (1.950-5.083)		2.937 (1.821-4.737)	
	Stage III	4.834 (2.998-7.796)		4.380 (2.722-7.048)	
	PTPN12 expression		<0.031		<0.001
	Low	2.352 (1.609-3.438)		2.114(1.449-3.084)	
	High	Reference		Reference	
SCC	pTNM Stage		0.014		0.009
	Stage I	Reference		Reference	
	Stage II	1.751 (0.852-3.595)		1.649(0.803-3.385)	
	Stage III	3.068 (1.449-6.495)		3.232 (1.524-6.856)	
	PTPN12 expression		0.056		0.104
	Low	1.200 (0.915-3.049)		1.650(0.903-3.015)	
	High	Reference		Reference	
Non-SCC	pTNM Stage		<0.001		<0.001
	Stage I	Reference		Reference	
	Stage II	4.909 (2.548-9.458)		4.401 (2.302-8.414)	
	Stage III	6.320 (3.334-11.980)		5.390 (2.864-10.142)	
	PTPNI2 expression		<0.001		<0.001
	Low	2.317 (1.438-3.798)		2.346(1.437-3.830)	
	High	Reference		Reference	

Abbreviation: PTPN12, tyrosine-protein phosph Mast non-receptor type 12; NSCLC, non-small cell lung cancer; SCC, squamous cell carcinoma; HR, hazard ratio; 95% CI, 95% confidence index.

a Cox proportional hazards model.

## DISCUSSION

Belonging to the PTPs family, PTPN12 is an enzyme that in humans is encoded by the *PTPN12* gene [13]. PTPN12 are known to be signaling molecules that regulate a variety of cellular processes [8, 9, 14, 15]. Researchers have recently suggested that PTPN12 may also be involved in the human carcinogenesis. For this reason, based on unique etiology, patient characteristics, uniform treatment modalities and long-term follow-up, the current study is the first to evaluate the expression and potential implications of PTPN12 in NSCLC patients. Notably, we observed that PTPN12 expression correlated significantly with visceral pleural invasion and pTNM stage. Most importantly, survival durations were significantly longer among patients with high-PTPN12 expression than among patients with low-PTPN12 expression. In the histology subgroup analyses, we observed that the survival benefit of high-PTPN12 expression might be limited in non-SCC patients. Our findings indicated that PTPN12 might act as a tumor suppressor and serve as a potential predictive biomarker for NSCLC patients, especially for those with non-SCC.

Some previous studies have reported that PTPs, including PTPN12, regulate the equilibrium of tyrosine phosphorylation and play a prominent role in tumor suppression [8-10, 16, 17]. Emma et al. demonstrated that *PTPN12* silencing enhances cell migration in ovarian cancer SKOV-3 cells, through focal adhesion kinase (FAK, a focal adhesion-associated protein kinase involved in cellular adhesion and spreading processes, and it often up-regulated in cancer cells [18-20] phosphorylation at Y397 [21]. Recently, Emma et al. carried out a laboratory investigation and found that silenced *PTPN12* depressed phosphatase and tensin homolog (PTEN) expression [22]. PTEN negatively regulates intracellular levels of phosphatidylinositol-3, 4, 5-trisphosphate in cells and functions as a tumor suppressor by negatively regulating PI3K/AKT signaling pathway [23-25]. This, in turn, contributes to suppress cell migration, through activation of GSK3 (target pS21-GSK3α) (GSK3, a substrate of AKT, a kinase regulating a number of diverse functions, including metabolism, cell cycle, cell migration and oncogenesis [26-28] and inhibition of FAK (target pS722) [22]. Furthermore, in the breast tumor MDA-MB-231 cells, PTPN12 silencing enhanced cell proliferation (unpublished results). Most of these findings support the function of PTPN12 as a tumor suppressor.

Recently, the study of Sun *et al*. demonstrated that loss of PTPN12 leads to morphogenesis and malignant transformation in mammary epithelial cells [12]. Interestingly, in breast cancer cells with loss of PTPN12 expression, restoring PTPN12 expression suppresses proliferation, tumorigenesis and metastasis. As well as, lung cells collected from dox-inducible *PTPN12* cDNA animals present significantly fewer metastases than dox-free animals (termed MDA-MB231-LM2 cells, a highly tumorigenic and metastatic subpopulation of MDA-MB231 [29, 30]. Collectively, Sun *et al*. have discovered a promising and extensive network of negative regulation with alternation oncogenes and tumor suppressors consisting of β-TRCP, REST, miR-214, and PTPN12, which inhibit RTKs to control cell proliferation, tumorigenesis, and survival.

Because of the crucial roles of PTPN12 in tumorigenesis, its potential application in the clinic has been evaluated. Along with pTNM stage, the expressive level of PTPN12 was shown to be an independent and significant prognostic parameter in resectable NSCLCs. Our previous investigation recently demonstrated significantly longer survival durations in esophageal cancer patients with high PTPN12 expression than those with low PTPN12 expression [31]. Although the population of our previous study limited to squamous cell carcinoma, our results suggested that PTPN12 might be served as a clinical predictive biomarker for various cancer populations. Wu *et al*. indicated that low PTPN12 expression is associated with poor prognosis and may be used as a potential prognostic variable in triple-negative breast cancer [32]. These findings were consistent with the present study showing a favorable prognostic impact of high PTPN12 expression.

We observed that the benefit of high-PTPN12 expression was especially significant in non-SCC patients. One explanation for this finding may be via inhibition of multiple RTKs. Adenocarcinoma, a subset of NSCLCs, have been shown to harbor activating mutations in the epidermal growth factor receptor gene (EGFR), such cancers are responsive to gefitinib, a specific inhibitor of the tyrosine kinase activity of EGFR. Sun and colleagues have indicated that PTPN12 suppresses tumorigenesis by inhibiting multiple RTKs. This is consistent with our study and illustrates a special role of PTPN12 according to histological type.

We acknowledged the limitations of the present retrospective analysis. Firstly, we did not examine the expressive level of the upstream and downstream effectors of PTPN12, which could provide accurate evidence of the functional status of PTPN12, further refine the prognostic role of this biomarker and potentially facilitate the establishment of patient-tailored medical strategies and supports. Secondly, our study population was composed of Chinese population, which somewhat limits the generalizability of our results. Accordingly, the results of further investigations including more diverse populations (white/black/brown participants) from other institutes are needed to confirm our findings. In addition, our study is a retrospective study, relied exclusively on a single-institutional database. In the future, we plan to expand the prospective analyses to include upstream and downstream effectors of PTPN12 in the samples of multi-institution, and results will be reported in future publications.

In conclusion, this is the first study to show that patients with completely resected NSCLCs and PTPN12 high expression tumors had favorable survival durations compared to that of patients with low PTPN12 expression. Our findings demonstrate that determination of PTPN12 expression in NSCLCs after radical operation can contribute as an independent predictor of patient survival. Further mechanistic studies will be vital to facilitate our understanding of PTPN12 functional role in NSCLC.

## PATIENTS AND METHODS

### Patients and treatment

From April 2002 and March 2005, at Sun Yat–Sen University Cancer Center, 240 patients with complete surgical resection (R0) for NSCLC were eligible for our study. Pretreatment evaluation included complete history and a physical examination, complete blood cell count, serum biochemistry, chest X-ray, computed tomography (CT) scans of the chest and abdomen, and bronchoscopy. Whole-body bones scan and magnetic resonance imaging (MRI) scan of brain were used to exclude possible metastasis. Patients who had previous malignant disease, a second primary tumor, suspected distant metastasis, non-curative resection (R1), died of postoperative complications/non-cancer related death and lost follow-up were excluded. All cases underwent complete resection of lung cancer with mediastinal lymph node dissection. Adjuvant chemotherapy (cisplatin-based combinations) was performed in patients with stage II and stage III disease if the patients could tolerate treatment after curative-intent surgery, or unless the patients refused adjuvant chemotherapy. Patients with stage III disease and pathologic evidence of N2 disease received postoperative mediastinal radiotherapy. Tumor differentiation grades were defined according to the World Health Organization criteria. Stage was recorded based on AJCC (2009) [33]. The study was approved by the medical ethics committee of the Cancer Center at Sun Yat-Sen University.

After completion of primary treatment, patients were followed up every 4–6 months in the first 3 years and every 12 months thereafter. The survival status was verified again using the best available methods in May 2010, including verifying the clinical attendance records and with direct telecommunication with the patient or their family.

### Construction of the tissue microarray

The tissue microarray was constructed according to methods described previously [34]. The tissues (240 NSCLC tissues and 50 paracancerous normal lung tissues) from the tumor bank were collected, fixed in ethanol, and embedded in paraffin [the paracancerous normal samples were obtained from the lung tissues which were: (1) 2cm or more; or (2) the size of the nodule or more from tumor margins; there was no microscopic residual tumor cells]. Hematoxylin and eosin–stained sections from a single random block from each patient were reviewed by a senior pathologist (Rong-Zhen Luo) to define representative tumor regions. Two targeted core samples of each specimen were obtained using a tissue array instrument (MiniCore instruments; Alphelys, Plaisir, France). Briefly, 10-mm tissue cylinders were punched and arrayed on a recipient paraffin block. Sections (5 μm) of the tissue array (recipient) block were cut and placed on glass slides.

### Western blot

Total proteins were extracted with 1×SDS sample buffer [62.5 mmol/L Tris-HCl (pH 6.8), 2% SDS, 10% glycerol, and 5% 2-mercaptoethanol]. The protein concentration was determined using the Bradford assay (Bio-Rad Laboratories, Hercules, CA). A total of 20μg protein was electrophoretically separated in 12% SDS polyacrylamide gels and transferred onto polyvinylidene difluoride membranes (Amersham Pharmacia Biotech, Piscataway, NJ). Anti-PTPN12 rabbit polyclonal (1:500, HPA007097, Prestige Antibodies, Atlas Antibodies, Sigma, more details: http://www.proteinatlas.org/ENSG00000127947-PTPN12/cancer) and anti-rabbit (1:2000, Santa Cruz Biotechnology, Santa Cruz, CA) antibodies were used to detect PTPN12 protein. Anti-α-tubulin mouse monoclonal (1:2000, Sigma) and anti-mouse (1:2000, Santa Cruz Biotechnology, Santa Cruz, CA) antibodies were used to confirm equal loading. The Western blot bands were scanned and analyzed by the Quantity One program (Bio-Rad, Hercules, CA).

### Immunohistochemistry

A standard protocol for the immunostaining of tissue microarray sections was used. In brief, tissue microarray sections were rehydrated through graded alcohol. Endogenous peroxidase activity was blocked with 0.3% hydrogen peroxide for 15 min. For epitope retrieval, the tissue microarray slides were exposed to 10 mM citrate buffer (pH 6.0) and heated for 5 min. The tissue microarray slides were incubated with anti-PTPN12 antibody at a dilution of 1:50 (HPA007097, 100 μl, Prestige Antibodies, Atlas Antibodies, Sigma, more details: http://www.proteinatlas.org/ENSG00000127947-PTPN12/cancer) for 12 hours at 4°C in a moist chamber. Subsequently, biotinylated secondary antibody was applied for 30 min at 37°C. Then, the sections were incubated with streptavidin–horseradish peroxidase complex and developed with 3-diaminobenzidine tetrahydrochloride (DAB). Mayer's hematoxylin was applied as a counterstain. PTPN12 immunopositive breast cancer slides were used as a positive control. As a negative control, the primary antibody was replaced by normal rabbit serum.

Cytoplasmic PTPN12 was evaluated according to the percentage of positively stained cells (median, 60%; range, 0-100%) and staining intensity (negative staining; low staining: light yellow; intermediate staining: yellow brown; and high staining: brown). PTPN12 expression index: 0: negative; 1: ≤60% of cells positive with low intensity; 2: > 60% of cells positive with low intensity or ≤ 60% with intermediate intensity; 3: > 60% of cells positive with intermediate intensity or ≤ 60% of cells positive with high intensity; and 4: > 60% of cells positive with high intensity. For PTPN12, the expression index of the paracancerous normal lung tissues was 2 to 4. Thus, we defined an expression index of 0 and 1 as the low expression, while an expression index of 2–4 was considered high expression.

Two investigators who were unaware of the clinicopathological data independently evaluated PTPN12 staining under a light microscope. In this study, a minimum of 300 epithelial cells was counted for each normal or tumor case. In order to reach a conclusive judgment, discordant cases were reviewed.

### Statistical analysis

The statistical analyses were performed using the SPSS 13.0 software package (SPSS, Inc., Chicago, IL). Disease-free survival (DFS) and overall survival (OS) were defined as the time from the date of surgery to the date of regional recurrence or distant metastasis and death or final clinical follow-up, respectively. The correlation between PTPN12 and clinicopathological characteristics was assessed using the χ^2^ test. Multivariate Cox regression analysis was performed for all parameters that were found to be significant by the univariate analysis. Actuarial survival rates were plotted against time using the Kaplan-Meier method, and log-rank testing was used to compare the differences between the curves. Two- sided p<0.05 was considered statistically significant.
